# Numerical study of diffusive fish farm system under time noise

**DOI:** 10.1038/s41598-024-62304-8

**Published:** 2024-06-26

**Authors:** Muhammad Waqas Yasin, Nauman Ahmed, Jawaria Saeed, Muhammad Zafarullah Baber, Syed Mansoor Ali, Ali Akgül, Shah Muhammad, Murad Khan Hassani, Mubasher Ali

**Affiliations:** 1 Department of Mathematics and Statistics, The University of Lahore, Lahore, Pakistan; 2 Department of Mathematics, University of Narowal, Narowal, Pakistan; 3 Department of Physics and Astronomy, College of Science, King Saud University, P.O. BOX 2455, 11451 Riyadh, Saudi Arabia; 4 Department of Mathematics, Art and Science Faculty, Siirt University, 56100 Siirt, Turkey; 5 Department of Computer Science and Mathematics, Lebanese American University, Beirut, Lebanon; 6 Department of Mathematics, College of Science, King Saud University, P.O. Box 2455, 11451 Riyadh, Saudi Arabia; 7 Department of Mathematics, Ghazni University, Ghazni, Afghanistan; 8 School of Engineering and Digital Arts, University of Kent, Canterbury, Kent UK

**Keywords:** Stochastic PDEs, Finite difference schemes, Analysis of schemes, Simulations, Engineering, Mathematics and computing, Physics

## Abstract

In the current study, the fish farm model perturbed with time white noise is numerically examined. This model contains fish and mussel populations with external food supplied. The main aim of this work is to develop time-efficient numerical schemes for such models that preserve the dynamical properties. The stochastic backward Euler (SBE) and stochastic Implicit finite difference (SIFD) schemes are designed for the computational results. In the mean square sense, both schemes are consistent with the underlying model and schemes are von Neumann stable. The underlying model has various equilibria points and all these points are successfully gained by the SIFD scheme. The SIFD scheme showed positive and convergent behavior for the given values of the parameter. As the underlying model is a population model and its solution can attain minimum value zero, so a solution that can attain value less than zero is not biologically possible. So, the numerical solution obtained by the stochastic backward Euler is negative and divergent solution and it is not a biological phenomenon that is useless in such dynamical systems. The graphical behaviors of the system show that external nutrient supply is the important factor that controls the dynamics of the given model. The three-dimensional results are drawn for the various choices of the parameters.

## Introduction

There are many industries in our surroundings. The waste of these industries is not pesticide-free matter, which pollutes the water body, ecosystem, and whole wildlife surrounding the water. Water is a good solvent that dissolves most substances and we believe that water can dilute or absorb any substance. Lakes, rivers, or any water body can be polluted due to pesticide wastage by industries^1^. Here, our intention is on wildlife like fish farms because fish farms completely depend on organic food and pesticide-free feeds. If the water is polluted then the outside food will be harmful to the fish^2^. Due to the excess use of natural foods, there will deficiency of oxygen in the water, and the harmful waste of industries draw inadequate effects on agriculture and wildlife like mussels, shellfish, oyster gulfweed, and rockweed. All these types of species are used as biomass, biofuel, or bio-filter that feed extra-undissolved substances in the water body^3^. As a result, water will purify and filter. These species have positive effects to purify the water and particularly shellfish have characteristics to filter the water, food, and feeds. Nowadays, many fishers use mussels, oysters, and shellfish as bio-filters or biomass to make highly purified fish farms. These can work in the following ways: one is, it consumes undissolved substances and fish waste, and other, it can feed pesticide and artificial food^4^. Many works are based on a system of differential equations that deal with such models of the population in mathematics and analyze their numerical results through different methods.

The disease dynamics are widely discussed due to the importance of human life and living organisms. Recently, the Coronavirus disturbed the human community drastically. Butt et al. used the optimal control analysis for the Corona disease^5^, considered the bi-model dynamics Covid-19 model, and analyzed the results by using optimal control theory^6^, used the optimal control strategies to eradicate the Rubella disease^7^, considered the fractional epidemic model with Atangana–Baleanu derivative^8^. Rafiq et al. proposed an Ebola epidemic model and analyzed its different aspects^9^. Butt et al. considered the Covid-19 pandemic model and used mathematical techniques such as the unique existence of the solution and other techniques to minimize the effect of the disease on the human community^10^. Hanif et al. employed the numerical technique to find the solution of the Caputo-Fabrizio-fractional model of the coronavirus pandemic^11^. Authors considered the various epidemic models analyzed the disease dynamics^12–14^.

A great number of stochastic process models investigated comprise space and spatial variables^15^. Nevertheless, a couple of authors studied and observed fish farm dynamics models just as stochastic procedures due to undetermined stochastic terms or variables^16^. In 1998, Virtala et al.^17^ constructed such a model, which is consistent and formulated the reason for dead fish due to inevitable accidents. In addition, from 1990 to 2003 Harris et al.^18^ worked on stochastic models which are of fault damage zones that are generated by statistical properties for fault populations. Yoshioka et al.^19^ showed the art of the state of modeling, computation, and analysis of stochastic control science in environmental engineering and research areas related to these fields. Gudmundsson et al.^20^ gave their efforts on an age-structured system comprised of time and space and described the gap connecting biofuel and the age of the system.

Furthermore, Sullivan et al.^21^ and Lewy et al.^22^ appreciate stochastic procedure in their models and contemplate it emphatically reliable method. Schnute^23^ narrated such models that accommodate a bit of error and then appraise both stochastic process and stochastic PDEs to estimate errors. Stochastic PDEs own such properties and characteristics that enhance unpredictability and errors. León-Santana et al.^24^ figure out procedures to accomplish earthly and habitat models based on linear stochastic PDEs. Likewise, Nøstbakken^25^ numerically works out on stochastic PDEs for ecological systems and wildlife like fish farms. Additionally, Reed and Clarke^26^ collected optimum reaping rules for organic phenomena along with stochastic PDEs. As our model is substantial and earthly so, we convert the system of PDEs into stochastic PDEs because the substantial model can perform randomness in behavior at some particular stage and results may not be predicted^27^. In extension, stochastic has numerous applications in real life. The majority of the researchers and authors used the stochastic in their work and paper. So, we prefer stochastic PDEs instead of simple PDEs^28^.

The fish form model under the influence of time noise is given as,

1 $$\begin{aligned} u_t= & {} d_u u_{xx}+\phi -\left( \mu +\alpha v+\xi w\right) u + \sigma _1 u\dot{B}_1(t), \end{aligned}$$

2 $$\begin{aligned} v_t= & {} d_v v_{xx} - \left( \delta + \gamma v-\beta u\right) v + \sigma _2 v\dot{B}_2(t), \end{aligned}$$

3 $$\begin{aligned} w_t= & {} d_w w_{xx} - \left( \rho - \eta u\right) w + \sigma _3 w\dot{B}_3(t), \end{aligned}$$

with initial conditions

4 $$\begin{aligned} u(x,0)= \alpha (x) \ge 0, v(x,0)= \beta (x) \ge 0, w(x,0)= \gamma (x) \ge 0, \end{aligned}$$

here *u*(*x*, *t*), *v*(*x*, *t*), and *w*(*x*, *t*) are the densities of nutrient, fish, and mussel population at a point (*x*, *t*) respectively. Also, there are four parameters present in the given model that is $\phi$, $\mu$, $\alpha$, and $\xi$ which describe the rate of external deposition of food, digestion rate of food of fish community and maximum digestion rate of food of mussel community respectively. These parameters have had a great effect on the ecosystem and its functioning. Here, $\delta$, $\gamma$, and $\beta$ describe the death rate respectively, competition occurring within a species, and the proportion of food supply to biofuel of fish. In addition, $\rho$ and $\eta$ describe the death rate and transfer the ability of mussel. Thus, the external deposition of food does not be digested into fish biofuel so that it could reach oysters in the form of specific pesticide-free matter and mussels can easily absorb it. Moreover, $d_u$, $d_v$ and $d_w$ be the coefficients of diffusion and $\dot{B}_i$ be the standard Wiener one-dimensional processes, with $\sigma _i$ that represent noisy strengths where $i=1,2,3$ and it is a Borel functions^29^.

To work on stochastic is a challenging task especially when we have to deal with non-linear terms. Numerous researchers worked on SPDEs and their numerical solutions by two finite difference schemes and methods and proved the consistency and stability^30^. The stochastic procedure can be beneficial to demonstrate some of the unpredictable results in the accomplishment of the distinct objective because they handle the randomness in the model^31^. A stochastic method commonly used in various fields and has real-life applications in gaming theory, surveys, tracking location, and probability statistical analysis^32^. In extension, SPDEs are used in numerous models such as the substantial or physical system with time frame because it consists of a random variable that calls noise term calculated with the Wiener process which dominates the unpredicted behavior of random behavior^33^. Therefore, it is extensively in numerous mathematical models like echography, pictorial representation, ultrasonography, computational molecular biology, and financial markets like a trading floor that vary with time randomly^34^.

Chessari et al. considered the backward stochastic differential equations and employed various numerical methods^35^. Zheng et al. used the finite elements methods for the study of SPDEs^36^. Röckner et al. worked on the wellposedness of the SPDEs^37^. Gyöngy et. al. used the lattice approximation of the SPDEs perturbed by white noise on a bounded domain $R^d$ for $d=1,2,3$ and gained the convergence rates of the approximation^38^. The authors gained the numerical approximation of Bagley-Trovik and fractional Painleve equations by using the cubic B-spline method^39^. Arqub et al. worked on the numerical computing of the Singular Lane-Emden type model by using the reproducing Kernal discretization method^40^. Sweilam et al. worked on the numerical solution of a stochastic extended Fisher-Kolmogorov equation perturbed by multiplicative noise^41^.

The main contribution of this work is given below:

- The classical models cannot represent the true behavior of nature. So, need of the hour to consider the classical model under the impact of some environmental noise.
- The fish farm model is considered under the temporal noise.
- The underlying is numerically investigated.
- Two schemes are constructed and used for the numerical study.
- Schemes are von Neumann stable and consistent.
- Equilibrium points are successfully gained.
- The graphical behavior of the state variables is explained from the biological point of view.
- The MATLAB 2015a is used for the graphical behavior of the test problem.

## Numerical methods

In order to analyze the given system of equations, we discretized the whole domain of space and temporal variables. The grid points $(x_l,t_m)$ are explained as

$$\begin{aligned} x_d= & {} dh, d=0,1,2,3,\ldots , M. \\ t_e= & {} ek, e=0,1,2,3,\ldots , N_1. \end{aligned}$$

Here, *M* and $N_1$ are the integers, and $\Delta x=h$ and $\Delta t= k$ are stepsizes of space and temporal respectively.

The proposed stochastic backward Euler(SBE) scheme of Eqs. (1), (2), (3) is

5 $$\begin{aligned}{} & {} (1+2\lambda _1)u_d^{e+1}-\lambda _1\left( u_{d+1}^{e+1}+u_{d-1}^{e+1}\right) = u_d^e -\Delta t\left( \mu + \alpha v_d^e +\xi w_d^e\right) u_d^e \nonumber \\{} & {} \quad + \Delta t \phi + \sigma _1 u_d^e \left( B_1^{(e+1)\Delta t} - B_1^{e\Delta t}\right) . \end{aligned}$$

6 $$\begin{aligned}{} & {} (1+2\lambda _2)v_d^{e+1}-\lambda _2\left( v_{d+1}^{e+1}+v_{d-1}^{e+1}\right) =v_d^e-\Delta t\left( \delta + \gamma v_d^e +\beta u_d^e\right) v_d^e \nonumber \\{} & {} \quad +\sigma _2 v_d^e \left( B_2^{(e+1)\Delta t} - B_2^{e\Delta t}\right) . \end{aligned}$$

7 $$\begin{aligned}{} & {} (1+2\lambda _3)w_d^{e+1}-\lambda _3\left( w_{d+1}^{e+1}+w_{d-1}^{e+1}\right) =w_d^e-\Delta t\left( \rho + \eta u_d^e \right) w_d^e \nonumber \\{} & {} \quad + \sigma _3 w_d^e \left( B_e3^{(e+1)\Delta t} - B_3^{e\Delta t}\right) . \end{aligned}$$

The Eqs. (5)–(7) are required proposed SBE scheme for Eqs. (1)–(3). The proposed stochastic Implicit finite difference (SIFD) scheme of Eqs. (1)–(3)

8 $$\begin{aligned}{} & {} \left( 1+2\lambda _1+\Delta t\left( \mu + \alpha v_d^e +\xi w_d^e\right) \right) u_d^{e+1}-\lambda _1\left( u_{d+1}^{e+1}+u_{d-1}^{e+1}\right) =u_d^e \nonumber \\{} & {} \quad + \Delta t \phi + \sigma _1 u_d^e \left( B_1^{(e+1)\Delta t} - B_1^{e\Delta t}\right) . \end{aligned}$$

9 $$\begin{aligned}{} & {} \bigg (1+2\lambda _2+\Delta t\left( \delta + \gamma v_d^e \right) \bigg ) v_d^{e+1}-\lambda _2\left( v_{d+1}^{e+1}+v_{d-1}^{e+1}\right) =v_d^e + \nonumber \\{} & {} \quad +\Delta t \beta u_d^e v_d^e+\sigma _2 v_d^e \left( B_2^{(e+1)\Delta t} - B_2^{e\Delta t}\right) . \end{aligned}$$

10 $$\begin{aligned}{} & {} (1+2\lambda _3+\Delta t\rho w_d^e )w_d^{e+1}-\lambda _3\left( w_{d+1}^{e+1}+w_{d-1}^{e+1}\right) =w_d^e+ \Delta t \eta u_d^e w_d^e \nonumber \\{} & {} \quad \times \sigma _3 w_d^e \left( B_e3^{(e+1)\Delta t} - B_3^{e\Delta t}\right) . \end{aligned}$$

The Eqs. (8)–(10) are required SIFD scheme for Eqs. (1)–(3).

Here $\lambda _1 = \frac{d_u\Delta t}{\Delta x^2}$ ,$\lambda _2 = \frac{d_v\Delta t}{\Delta x^2}$, $\lambda _3 = \frac{d_w\Delta t}{\Delta x^2}$. The numerical approximation of *u*(*x*, *t*), *v*(*x*, *t*) and *w*(*w*, *t*) is $u_d^e, v_d^e, w_d^e$ at point $(d \Delta x,e \Delta t)$. The analysis of the scheme is discussed in the next sections.

## Stability analysis

By Von-Neuman method of stability^42^,

11 $$\begin{aligned} A_m^n=\frac{1}{\sqrt{2\pi }}\int _{\frac{-\pi }{\Delta x}}^{\frac{\pi }{\Delta x}}e^{im\Delta x \theta } \hat{A}_m^n(\theta )d(\theta ), \end{aligned}$$

where $\hat{A}^n_m$ is a Fourier transformation of $A^n_m$ which is given below,

12 $$\begin{aligned} {\hat{A}}_m^n=\frac{1}{\sqrt{2\pi }}\sum _{-\infty }^{\infty } e^{im\Delta x \theta } A_m^n \Delta x. \end{aligned}$$

Where $\theta$ is a real variable. By substituting this value in finite schemes, we may get

$$\begin{aligned} \hat{u}^{(e+1)}(\theta ) =\hat{u}^e(\theta )g(\theta \Delta x, \Delta \tau , \Delta x). \end{aligned}$$

The sufficient and necessary condition for Von Neumann the stability method is,

$$\begin{aligned} E|g(\theta \Delta x, \Delta \tau , \Delta x)|^2 \le 1+ \varrho \Delta \tau . \end{aligned}$$

where $\varrho$ is constant.

### Theorem 1

The SBE scheme for *u*(*x*, *t*), *v*(*x*, *t*), and *w*(*x*, *t*) is von Neumann stable in the mean square sense.

### Proof

Von Neuman stability criteria are used for linear equations. So non-Linear term can be linearized by taking $v_d^e= \psi _1$ and $w_d^e=\psi _2$ where $\psi _1$, $\psi _2$ and $\phi$ are local constants so it can be set equal to zero. Thus, finite difference scheme for Eqs. (1) in (5) can be written as,

13 $$\begin{aligned}{} & {} (1+2\lambda _1)u_d^{e+1}-\lambda _1\left( u_{d+1}^{e+1}+u_{d-1}^{e+1}\right) =u_d^e -\Delta t \mu u_d^e + \sigma _1 u_d^e ( B_1^{(e+1)\Delta t} - B_1^{e\Delta t}), \nonumber \\{} & {} \frac{1}{\sqrt{2\pi }}\int _{\frac{-\pi }{\Delta x}}^{\frac{\pi }{\Delta x}}e^{id\Delta x \theta } \bigg (1+2\lambda _1 -\lambda _1 (e^{i\Delta x \theta }+e^{-i\Delta x \theta } )\bigg )\hat{u}^{(e+1)}(\theta )d(\theta ) \nonumber \\{} & {} \quad =\frac{1}{\sqrt{2\pi }}\int _{\frac{-\pi }{\Delta x}}^{\frac{\pi }{\Delta x}}e^{id\Delta x \theta } \bigg (1 -\Delta t \mu + \sigma _1( B_1^{(e+1)\Delta t} - B_1^{e\Delta t})\bigg ) \hat{u}^e(\theta )d(\theta ). \nonumber \\{} & {} \left( 1+2\lambda _1 - 2\lambda _1 +4\lambda _1 \sin ^2 \frac{\Delta x \theta }{2}\right) \hat{u}^{(e+1)}(\theta )= \bigg (1 -\Delta t \mu + \sigma _1( B_1^{(e+1)\Delta t} - B_1^{e\Delta t})\bigg )\hat{u}^e(\theta ). \nonumber \\{} & {} \quad \frac{ \hat{u}^{(e+1)}(\theta )}{\hat{u}^e(\theta )}= \frac{1 -\Delta t \mu + \sigma _1( B_1^{(e+1)\Delta t} - B_1^{e\Delta t})}{1+4\lambda _1 \sin ^2 \frac{\Delta x \theta }{2}}. \end{aligned}$$

By amplification factor, Eq. (13) can be written as,

14 $$\begin{aligned} g_1(\theta \Delta x, \Delta t, \Delta x)= \frac{1 -\Delta t \mu + \sigma _1( B_1^{(e+1)\Delta t} - B_1^{e\Delta t})}{1+4\lambda _1 \sin ^2 \frac{\Delta x \theta }{2}}. \end{aligned}$$

Now by using independent of Wiener process increment and amplification factor, we reached at

15 $$\begin{aligned} E|g_1(\theta \Delta x, \Delta t, \Delta x)|^2 \le \left| \frac{1 -\Delta t \mu }{ 1+4\lambda _1 \sin ^2\frac{\Delta x \theta }{2}} \right| ^2 + \left| \frac{\sigma _1}{ 1+4\lambda _1 \sin ^2 \frac{\Delta x \theta }{2}}\right| ^2 \Delta t. \end{aligned}$$

$$\left| \frac{1 -\Delta t \mu }{\left( 1+4\lambda _1 \sin ^2 \frac{\Delta x \theta }{2}\right) } \right| ^2\le 1$$, then (15) becomes,

16 $$\begin{aligned} E|g_1(\theta \Delta x, \Delta t, \Delta x)|^2 \le 1+ \varrho _1 \Delta t. \end{aligned}$$

Here, $\left| \frac{\sigma _1}{\left( 1+4\lambda _1 \sin ^2 \frac{\Delta x \theta }{2}\right) }\right| ^2 = \varrho _1$. According to the stability definition, we deduced that Eq. (1)is von Neumann stable.

Similarly, Eq. (6) is linearized as follows,

17 $$\begin{aligned}{} & {} (1+2\lambda _2)v_d^{e+1}-\lambda _2\left( v_{d+1}^{e+1}+v_{d-1}^{e+1}\right) =v_d^e-\Delta t \delta v_d^e + \sigma _2 v_d^e \left( B_2^{(e+1)\Delta t} - B_2^{e\Delta t}\right) . \nonumber \\{} & {} \frac{1}{\sqrt{2\pi }}\int _{\frac{-\pi }{\Delta x}}^{\frac{\pi }{\Delta x}}e^{id\Delta x \theta } \bigg (1+2\lambda _2 -\lambda _2 (e^{i\Delta x \theta }+e^{-i\Delta x \theta })\bigg )\hat{v}^{(e+1)}(\theta )d(\theta ) \nonumber \\{} & {} \quad = \frac{1}{\sqrt{2\pi }}\int _{\frac{-\pi }{\Delta x}}^{\frac{\pi }{\Delta x}}e^{id\Delta x \theta } \bigg (1-\Delta t \delta + \sigma _2( B_2^{(e+1)\Delta t} - B_2^{e\Delta t})\bigg ) \hat{v}^e(\theta )d(\theta ). \nonumber \\{} & {} \left( 1+2\lambda _2 -2\lambda _2 +4 \lambda _2 \sin ^2 \frac{\Delta x \theta }{2}\right) \hat{v}^{(e+1)}(\theta )= (1-\Delta t\delta + \sigma _2( B_2^{(e+1)\Delta t} - B_2^{e\Delta t})) \hat{v}^e(\theta ). \end{aligned}$$

18 $$\begin{aligned}{} & {} \frac{\hat{v}^{(e+1)}(\theta )}{\hat{v}^e(\theta )}= \frac{1-\Delta t \delta + \sigma _2( B_2^{(e+1)\Delta t} - B_2^{e\Delta t})}{1+ 4 \lambda _2 \sin ^2 \frac{\Delta x \theta }{2}}. \end{aligned}$$

19 $$\begin{aligned}{} & {} \quad g_2(\theta \Delta x, \Delta t, \Delta x)= \frac{1-\Delta t \delta + \sigma _2( B_2^{(e+1)\Delta t} - B_2^{e\Delta t})}{1+ 4 \lambda _2 \sin ^2 \frac{\Delta x \theta }{2}}. \end{aligned}$$

Now by using independent of Wiener process increment and amplification factor, we reached at

20 $$\begin{aligned} E|g_2(\theta \Delta x, \Delta t, \Delta x)|^2 \le \left| \frac{1-\Delta t \delta }{1+ 4 \lambda _2 \sin ^2 \frac{\Delta x \theta }{2}}\right| ^2 +\left| \frac{\sigma _2 }{1+ 4 \lambda _2 \sin ^2 \frac{\Delta x \theta }{2}}\right| ^2\Delta t. \end{aligned}$$

$\left| \frac{1 -\Delta t \delta }{\left( 1+4\lambda _1 \sin ^2 \frac{\Delta x \theta }{2}\right) } \right| ^2\le 1$, then equation (20) becomes,

21 $$\begin{aligned} E|g_2(\theta \Delta x, \Delta t, \Delta x)|^2 \le 1+ \varrho _2 \Delta t. \end{aligned}$$

Here, $\left| \frac{\sigma _2}{\left( 1+4\lambda _1 \sin ^2 \frac{\Delta x \theta }{2}\right) }\right| ^2 = \varrho _2$. According to the stability definition, we deduced that this scheme is von Neumann stable.

Similarly, Eq. (7) is linearized as follows,

22 $$\begin{aligned}{} & {} (1+2\lambda _3)w_d^{e+1}-\lambda _3\left( w_{d+1}^{e+1}+w_{d-1}^{e+1}\right) =w_d^e-\Delta t\rho w_d^e + \sigma _3 w_d^e \left( B_e3^{(e+1)\Delta t} - B_3^{e\Delta t}\right) . \nonumber \\{} & {} \frac{1}{\sqrt{2\pi }}\int _{\frac{-\pi }{\Delta x}}^{\frac{\pi }{\Delta x}}e^{id\Delta x \theta }\bigg (1+2\lambda _3 -\lambda _3 (e^{i\Delta x \theta }+e^{-i\Delta x \theta })\bigg )\hat{w}^{(e+1)}(\theta )d(\theta ) \nonumber \\{} & {} \quad =\frac{1}{\sqrt{2\pi }}\int _{\frac{-\pi }{\Delta x}}^{\frac{\pi }{\Delta x}}e^{id\Delta x \theta } \bigg (1-\Delta t \rho + \sigma _3( B_3^{(e+1)\Delta t} - B_3^{e\Delta t})\bigg ) \hat{w}^e(\theta )d(\theta ). \nonumber \\{} & {} \left( 1+2\lambda _3 \right) -\lambda _3 (e^{i\Delta x \theta }+e^{-i\Delta x \theta })\hat{w}^{(e+1)}(\theta ) = \bigg (1-\Delta t \rho + \sigma _3( B_3^{(e+1)\Delta t} - B_3^{e\Delta t})\bigg ) \hat{w}^e(\theta ). \nonumber \\{} & {} \left( 1+2\lambda _3 -2\lambda _3 + 4 \lambda _3 \sin ^2\frac{\Delta x \theta }{2}\right) \hat{w}^{(e+1)}(\theta ) = \bigg (1-\Delta t \rho + \sigma _3( B_3^{(e+1)\Delta t} - B_3^{e\Delta t})\bigg ) \hat{w}^e(\theta ). \end{aligned}$$

23 $$\begin{aligned}{} & {} \frac{\hat{w}^{(e+1)}(\theta )}{\hat{w}^e(\theta )}= \frac{1-\Delta t \rho + \sigma _3( B_3^{(e+1)\Delta t} - B_3^{e\Delta t})}{1+4 \lambda _3 \sin ^2 \frac{\Delta x \theta }{2}}. \end{aligned}$$

24 $$\begin{aligned}{} & {} g_3(\theta \Delta x, \Delta t, \Delta x)= \frac{1-\Delta t \rho + \sigma _3( B_3^{(e+1)\Delta t} - B_3^{e\Delta t})}{1+4 \lambda _3 \sin ^2\frac{\Delta x \theta }{2}}. \end{aligned}$$

Now by using independent of Wiener process increment and amplification factor, we reached at

25 $$\begin{aligned} E|g_3(\theta \Delta x, \Delta t, \Delta x)|^2 \le \left| \frac{1-\Delta t \rho }{1+ 4 \lambda _3 \sin ^2 \frac{\Delta x \theta }{2}}\right| ^2 +\left| \frac{\sigma _3 }{1+ 4 \lambda _3 \sin ^2 \frac{\Delta x \theta }{2}}\right| ^2\Delta t. \end{aligned}$$

$$\left| \frac{1 -\Delta t \rho }{\left( 1+4\lambda _3 \sin ^2 \frac{\Delta x \theta }{2}\right) } \right| ^2\le 1$$, then (25) becomes,

26 $$\begin{aligned} E|g_3(\theta \Delta x, \Delta t, \Delta x)|^2 \le 1+ \varrho _3 \Delta t. \end{aligned}$$

Here, $\left| \frac{\sigma _3}{\left( 1+4\lambda _3 \sin ^2 \frac{\Delta x \theta }{2}\right) }\right| ^2 = \varrho _3$. According to the stability definition, we deduced that this scheme is von Neumann stable. 

### Theorem 2

The SIFD scheme for *u*(*x*, *t*), *v*(*x*, *t*), and *w*(*x*, *t*) is von Neumann stable in the mean square sense.

### Proof

Similarly, Eq. (8) is linearized as follows,

27 $$\begin{aligned}{} & {} \left( 1+2\lambda _1+\Delta t\mu \right) u_d^{e+1}-\lambda _1\left( u_{d+1}^{e+1}+u_{d-1}^{e+1}\right) =u_d^e + \sigma _1 u_d^e \left( B_1^{(e+1)\Delta t} - B_1^{e\Delta t}\right) . \nonumber \\{} & {} \frac{1}{\sqrt{2\pi }}\int _{\frac{-\pi }{\Delta x}}^{\frac{\pi }{\Delta x}}e^{id\Delta x \theta }\bigg (1+2\lambda _1+\Delta t\mu -\lambda _1 (e^{i\Delta x \theta }+e^{-i\Delta x \theta })\bigg )\hat{u}^{(e+1)}(\theta )d(\theta )\nonumber \\{} & {} \quad =\frac{1}{\sqrt{2\pi }}\int _{\frac{-\pi }{\Delta x}}^{\frac{\pi }{\Delta x}}e^{id\Delta x \theta }\bigg (1 + \sigma _1( B_1^{(e+1)\Delta t} - B_1^{e\Delta t})\bigg )\hat{u}^e(\theta )d(\theta ). \nonumber \\{} & {} \left( 1+2\lambda _1+\Delta t \mu -2 \lambda _1+ 4\lambda _1 \sin ^2 \frac{\Delta x \theta )}{2}\right) \hat{u}^{(e+1)}(\theta )= (1 + \sigma _1( B_1^{(e+1)\Delta t} - B_1^{e\Delta t}))\hat{u}^e(\theta ). \end{aligned}$$

28 $$\begin{aligned}{} & {} \frac{\hat{u}^{(e+1)}(\theta )}{\hat{u}^e(\theta )}= \frac{1 + \sigma _1( B_1^{(e+1)\Delta t} - B_1^{e\Delta t})}{1+\Delta t \mu + 4\lambda _1 \sin ^2 \frac{\Delta x \theta }{2}}. \end{aligned}$$

29 $$\begin{aligned}{} & {} g_4(\theta \Delta x, \Delta t, \Delta x)= \frac{1 + \sigma _1( B_1^{(e+1)\Delta t} - B_1^{e\Delta t})}{1+\Delta t \mu + 4\lambda _1 \sin ^2 \frac{\Delta x \theta }{2}}. \end{aligned}$$

Now by using independent of Wiener process increment and amplification factor, we reached at

30 $$\begin{aligned} E|g_4(\theta \Delta x, \Delta \tau , \Delta x)|^2 \le \left| \frac{1}{1+\Delta t \mu + 4\lambda _1 \sin ^2 \frac{\Delta x \theta }{2}}\right| ^2 +\left| \frac{\sigma _1 }{1+\Delta t \mu + 4\lambda _1 \sin ^2 \frac{\Delta x \theta }{2}}\right| ^2\Delta t. \end{aligned}$$

$$\left| \frac{1}{1+\Delta t \mu + 4\lambda _1 \sin ^2 \frac{\Delta x \theta }{2}} \right| ^2\le 1$$, then (30) becomes,

31 $$\begin{aligned} E|g_4(\theta \Delta x, \Delta t, \Delta x)|^2 \le 1+ \varrho _4 \Delta t. \end{aligned}$$

Here, $\left| \frac{\sigma _1}{1+\Delta t \mu + 4\lambda _1 \sin ^2 \frac{\Delta x \theta }{2}}\right| ^2 = \varrho _4$. According to the stability definition, we deduced that (8) is von Neumann stable.

Similarly, Eq. (9) is linearized as follows,

32 $$\begin{aligned}{} & {} (1+2\lambda _2+\Delta t\delta )v_d^{e+1}-\lambda _2\left( v_{d+1}^{e+1}+v_{d-1}^{e+1}\right) =v_d^e +\sigma _2 v_d^e \left( B_2^{(e+1)\Delta t} - B_2^{e\Delta t}\right) . \nonumber \\{} & {} \left( 1+2\lambda _2+\Delta t\delta \right) \frac{1}{\sqrt{2\pi }}\int _{\frac{-\pi }{\Delta x}}^{\frac{\pi }{\Delta x}}e^{id\Delta x \theta } \hat{v}^{(e+1)}(\theta )d(\theta ) -\lambda _2\bigg (\frac{1}{\sqrt{2\pi }}\int _{\frac{-\pi }{\Delta x}}^{\frac{\pi }{\Delta x}}e^{i(d+1)\Delta x \theta } \hat{v}^{(e+1)}(\theta )d(\theta ) \nonumber \\{} & {} \qquad +\frac{1}{\sqrt{2\pi }}\int _{\frac{-\pi }{\Delta x}}^{\frac{\pi }{\Delta x}}e^{i(d-1)\Delta x \theta } \hat{v}^{(e+1)} (\theta )d(\theta )\bigg ) =\frac{1}{\sqrt{2\pi }}\int _{\frac{-\pi }{\Delta x}}^{\frac{\pi }{\Delta x}}e^{id\Delta x \theta } \hat{v}^e(\theta )d(\theta ) \nonumber \\{} & {} \qquad + \sigma _2 \frac{1}{\sqrt{2\pi }}\int _{\frac{-\pi }{\Delta x}}^{\frac{\pi }{\Delta x}}e^{id\Delta x \theta } \left( B_2^{(e+1)\Delta t} - B_2^{e\Delta t}\right) \hat{v}^e(\theta )d(\theta ). \nonumber \\{} & {} \frac{1}{\sqrt{2\pi }}\int _{\frac{-\pi }{\Delta x}}^{\frac{\pi }{\Delta x}}e^{id\Delta x \theta } \bigg (1+2\lambda _2+\Delta t\delta -\lambda _2 (e^{i\Delta x \theta }+e^{-i\Delta x \theta })\bigg )\hat{v}^{(e+1)}(\theta )d(\theta ) \nonumber \\{} & {} \quad =\frac{1}{\sqrt{2\pi }}\int _{\frac{-\pi }{\Delta x}}^{\frac{\pi }{\Delta x}}e^{id\Delta x \theta } \bigg (1+ \sigma _2( B_2^{(e+1)\Delta t} - B_2^{e\Delta t})\bigg ) \hat{v}^e(\theta )d(\theta ). \nonumber \\{} & {} \bigg (1+2\lambda _2+\Delta t\delta -\lambda _2 (e^{i\Delta x \theta }+e^{-i\Delta x \theta })\bigg )\hat{v}^{(e+1)}(\theta )= \bigg (1+ \sigma _2( B_2^{(e+1)\Delta t} - B_2^{e\Delta t})\bigg ) \hat{v}^e(\theta ). \nonumber \\{} & {} \left( 1+2\lambda _2+\Delta t\delta -2\lambda _2 +4\lambda _2 \sin ^2 \frac{\Delta x \theta }{2}\right) \hat{v}^{(e+1)}(\theta )= (1+ \sigma _2( B_2^{(e+1)\Delta t} - B_2^{e\Delta t})) \hat{v}^e(\theta ). \end{aligned}$$

33 $$\begin{aligned}{} & {} \frac{\hat{v}^{(e+1)}(\theta )}{\hat{v}^e(\theta )}= \frac{1+ \sigma _2( B_2^{(e+1)\Delta t} - B_2^{e\Delta t})}{\left( 1+\Delta t\delta +4\lambda _2 \sin ^2\frac{\Delta x \theta }{2}\right) }. \end{aligned}$$

34 $$\begin{aligned}{} & {} g_5(\theta \Delta x, \Delta t, \Delta x)= \frac{1+ \sigma _2( B_2^{(e+1)\Delta t} - B_2^{e\Delta t})}{\left( 1+\Delta t\delta +4\lambda _2 \sin ^2 \frac{\Delta x \theta }{2}\right) }. \end{aligned}$$

Now by using independent of Wiener process increment and amplification factor, we reached at

35 $$\begin{aligned} E|g_5(\theta \Delta x, \Delta t, \Delta x)|^2 \le \left| \frac{1}{1+\Delta t\delta +4\lambda _2 \sin ^2 \frac{\Delta x \theta }{2}}\right| ^2+\left| \frac{ \sigma _2}{\left( 1+\Delta t\delta +4\lambda _2 \sin ^2 \frac{\Delta x \theta }{2}\right) }\right| ^2\Delta t. \end{aligned}$$

$\left| \frac{1}{1+\Delta t\delta +4\lambda _2 \sin ^2 \frac{\Delta x \theta }{2}} \right| ^2\le 1$, then Eq. (35) becomes,

36 $$\begin{aligned} E|g_5(\theta \Delta x, \Delta t, \Delta x)|^2 \le 1+ \varrho _5 \Delta t. \end{aligned}$$

Here, $\left| \frac{\sigma _2}{1+\Delta t\delta +4\lambda _2 \sin ^2 \frac{\Delta x \theta }{2}}\right| ^2 = \varrho _5$. According to the stability definition, we deduced that (9)is von Neumann stable.

Similarly, Eq. (10) is linearized as follows,

37 $$\begin{aligned}{} & {} (1+2\lambda _3+\Delta t\rho )w_d^{e+1}-\lambda _3\left( w_{d+1}^{e+1}+w_{d-1}^{e+1}\right) =w_d^e+ \sigma _3 w_d^e \left( B_3^{(e+1)\Delta t} - B_3^{e\Delta t}\right) . \nonumber \\{} & {} \left( 1+2\lambda _3+\Delta t\rho \right) \frac{1}{\sqrt{2\pi }}\int _{\frac{-\pi }{\Delta x}}^{\frac{\pi }{\Delta x}}e^{id\Delta x \theta } \hat{w}^{(e+1)}(\theta )d(\theta ) -\lambda _3\bigg (\frac{1}{\sqrt{2\pi }}\int _{\frac{-\pi }{\Delta x}}^{\frac{\pi }{\Delta x}}e^{i(d+1)\Delta x \theta } \hat{w}^{(e+1)}(\theta )d(\theta ) \nonumber \\{} & {} \qquad +\frac{1}{\sqrt{2\pi }}\int _{\frac{-\pi }{\Delta x}}^{\frac{\pi }{\Delta x}}e^{i(d-1)\Delta x \theta } \hat{w}^{(e+1)} (\theta )d(\theta )\bigg ) =\frac{1}{\sqrt{2\pi }}\int _{\frac{-\pi }{\Delta x}}^{\frac{\pi }{\Delta x}}e^{id\Delta x \theta } \hat{w}^e(\theta )d(\theta ) \nonumber \\{} & {} \qquad + \sigma _3 \frac{1}{\sqrt{2\pi }}\int _{\frac{-\pi }{\Delta x}}^{\frac{\pi }{\Delta x}}e^{id\Delta x \theta } \bigg ( B_3^{(e+1)\Delta t} - B_3^{e\Delta t}\bigg )\hat{w}^e(\theta )d(\theta ). \nonumber \\{} & {} \frac{1}{\sqrt{2\pi }}\int _{\frac{-\pi }{\Delta x}}^{\frac{\pi }{\Delta x}}e^{id\Delta x \theta }\left( 1+2\lambda _3+\Delta t\rho \right) -\lambda _3 (e^{i\Delta x \theta }+e^{-i\Delta x \theta })\hat{w}^{(e+1)}(\theta )d(\theta ) \nonumber \\{} & {} \quad =\frac{1}{\sqrt{2\pi }}\int _{\frac{-\pi }{\Delta x}}^{\frac{\pi }{\Delta x}}e^{id\Delta x \theta } (1+ \sigma _3( B_3^{(e+1)\Delta t} - B_3^{e\Delta t})) \hat{w}^e(\theta )d(\theta ). \nonumber \\{} & {} \left( 1+2\lambda _3+\Delta t\rho \right) -\lambda _3 (e^{i\Delta x \theta }+e^{-i\Delta x \theta })\hat{w}^{(e+1)}(\theta )=(1+ \sigma _3( B_3^{(e+1)\Delta t} - B_3^{e\Delta t})) \hat{w}^e(\theta ). \nonumber \\{} & {} \left( 1+2\lambda _3+\Delta t \rho -2 \lambda _3 + 4\lambda _3 \sin ^2 \frac{\Delta x \theta }{2}\right) \hat{w}^{(e+1)}(\theta ) =(1+ \sigma _3( B_3^{(e+1)\Delta t} - B_3^{e\Delta t})) \hat{w}^e(\theta ). \nonumber \\{} & {} \frac{\hat{w}^{(e+1)}(\theta )}{\hat{w}^e(\theta )} =\frac{(1+ \sigma _3( B_3^{(e+1)\Delta t} - B_3^{e\Delta t}))}{\left( 1 +\Delta t\rho +4\lambda _3 \sin ^2 \frac{\Delta x \theta }{2}\right) }. \end{aligned}$$

38 $$\begin{aligned}{} & {} g_6(\theta \Delta x, \Delta t, \Delta x)= \frac{1+ \sigma _3( B_3^{(e+1)\Delta t} - B_3^{e\Delta t})}{1+\Delta t\rho +4\lambda _3 \sin ^2\frac{\Delta x \theta }{2}}. \end{aligned}$$

Now by using independent of Wiener process increment and amplification factor, we reached at

39 $$\begin{aligned} E|g_6(\theta \Delta x, \Delta t, \Delta x)|^2 \le \left| \frac{1}{1+\Delta t\rho +4\lambda _3 \sin ^2 \frac{\Delta x \theta }{2}}\right| ^2+\left| \frac{ \sigma _3 }{1+\Delta t\rho +4\lambda _3 \sin ^2 \frac{\Delta x \theta }{2}}\right| ^2\Delta t. \end{aligned}$$

$\left| \frac{1}{1+\Delta t\rho +4\lambda _3 \sin ^2 \frac{\Delta x \theta }{2}} \right| ^2\le 1$, then equation(39) becomes,

40 $$\begin{aligned} E|g_6(\theta \Delta x, \Delta t, \Delta x)|^2 \le 1+ \varrho _6 \Delta t. \end{aligned}$$

Here, $\left| \frac{\sigma _3}{1+\Delta t\rho +4\lambda _3 \sin ^2\frac{\Delta x \theta }{2}}\right| ^2 = \varrho _6$. According to the stability definition, we deduced that equation(10)is von Neumann stable.

## Consistency

A finite difference scheme $L|_{(l,m)} u|_{(l,m)} = G|_{(l,m)}$ is consistent with SPDE $LU=G$ at a point (*x*, *t*), if for any continuously differentiable function $\phi (x,t) = \phi$ in mean square that is

$E||(L\phi -G)|_{(l,m)}- [L|_{(l,m)}\phi |_{(lh, mk)}-G(l,m)]||^2 \rightarrow 0$, as $h \rightarrow 0$, $k\rightarrow 0$ and $(lh, (m+1)k)$ approaches to (*x*, *t*).

### Theorem 3

The SBE scheme given in Eqs. (5)–(7) is consistent in the mean square with SPDE Eqs. (1)–(3).

### Proof

The consistency of the proposed SBE scheme is given as By using the operator as $F(u)=\int _{mk}^{(m+1)k} u dr$ on Eq. (1)

41 $$\begin{aligned} F(u)|_d^e= & {} \int _{ek}^{(e+1)k} u(dh, r) dr - \int _{ek}^{(e+1)k} \phi dr - d_u \int _{ek}^{(e+1)k} u{xx}(dh, r) dr \nonumber \\{} & {} \quad + \int _{ek}^{(e+1)k} \left( \mu +\alpha v(dh, r)+ \xi w(dh, r) \right) u(dh, r)dr \nonumber \\{} & {} \quad - \sigma _1 \int _{ek}^{(e+1)k} u(dh, r) dB_1(r). \end{aligned}$$

42 $$\begin{aligned} F(u)|_d^e= & {} u{(dh, (e+1)k)}- u{(dh, ek)}- \int _{ek}^{(e+1)k} \phi dr - d_u \int _{ek}^{(e+1)k} u_{xx}(dh, r) dr \nonumber \\{} & {} \quad + \int _{ek}^{(e+1)k} \left( \mu +\alpha v{(dh, r)}+ \xi w{(dh, r)} \right) u{(dh, r)}dr \nonumber \\{} & {} \quad - \sigma _1 \int _{ek}^{(e+1)k} u{(dh, r)} dB_{1}(r). \end{aligned}$$

43 $$\begin{aligned} F|_d^e(u)= & {} u{(dh, (e+1)k)}- u{(dh, ek)}-k\phi - d_u k u_{xx}(dh, k) \nonumber \\{} & {} \quad + k \left( \mu +\alpha v{(dh, k)}+ \xi w{(dh, k)} \right) u{(dh, k)} \nonumber \\{} & {} \quad - \sigma _1 k u{(dh, k)} dB_{1}(k). \nonumber \\ F|_d^e(u)= & {} u{(dh, (e+1)k)}- u{(dh, ek)}-k \phi \end{aligned}$$

44 $$\begin{aligned}{} & {} \quad - d_u k \left( \frac{u{\left( (d+1)h,(e+1)k\right) }-2u{(dh,(e+1)k)}+u{\left( (d-1)h,(e+1)k \right) }}{h^2} \right) \nonumber \\{} & {} \quad + k \left( \mu +\alpha v{(dh, ek)}+ \xi w{(dh, ek)} \right) u{(dh, ek)}\nonumber \\{} & {} \quad - \sigma _1 ku{(dh, ek)} dB_{1k}. \end{aligned}$$

Hence, we conclude that

45 $$\begin{aligned}{} & {} E|F(u)|_d^e -F|_d^e(u)|^2 \nonumber \\{} & {} \quad \le 3(\sigma _1)^2 E\left| \int _{ek}^{(e+1)k} \left( -u{(dh, r)} +u{(dh, ek)} \right) dB_{1r}\right| ^2 \nonumber \\{} & {} \qquad +3d_u^2 E\left| \int _{ek}^{(e+1)k} \left( -u_{xx}(dh, r)+ \frac{u{\left( (d+1)h,(e+1)k \right) }-2u{(dh,(e+1)k)}+u{\left( (d-1)h,(e+1)k \right) }}{h^2} \right) dr\right| ^2 \nonumber \\{} & {} \qquad + 3E\left| \int _{ek}^{(e+1)k}\left( \left( \mu +\alpha v{(dh, r)}+ \xi w{(dh, r)} \right) u{(dh, r)}-\left( \mu +\alpha v{(dh, ek)}+ \xi w{(dh,ek)} \right) u{(dh, ek)}\right) dr\right| ^2. \end{aligned}$$

Now the square of It$\hat{o}$ Integral gives us

46 $$\begin{aligned}{} & {} E|F(u)|_d^e -F|_d^e(u)|^2 \nonumber \\{} & {} \quad \le 3(\sigma _1)^2 \int _{ek}^{(e+1)k} E\left| -u{(dh, r)} +u{(dh, ek)}\right| ^2 dr \nonumber \\{} & {} \qquad +3d_u^2 E\left| \int _{ek}^{(e+1)k} \left( -u_{xx}(dh, r)+ \frac{u{\left( (d+1)h,(e+1)k \right) }-2u{(dh,(e+1)k)}+u{\left( (d-1)h,(e+1)k \right) }}{h^2} \right) dr\right| ^2 \nonumber \\{} & {} \qquad + 3E\left| \int _{ek}^{(e+1)k}\left( \left( \mu +\alpha v{(dh, r)}+ \xi w{(dh, r)} \right) u{(dh, r)}-\left( \mu +\alpha v{(dh, ek)}+ \xi w{(dh,ek)} \right) u{(dh, ek)}\right) dr\right| ^2. \end{aligned}$$

$E|F(u)|_d^e -F|_d^e(u)|^2\rightarrow 0$ as $(d,e)\rightarrow \infty$. So, the given scheme for state variable *u* is consistent.

Similarly, Consistency for Eq. (6) is as follows,

47 $$\begin{aligned} F(v)|_d^e= & {} v{(dh, (e+1)k)}- v{(dh, ek)} - d_v \int _{ek}^{(e+1)k} v_{xx}(dh, r) dr \nonumber \\{} & {} \quad + \int _{ek}^{(e+1)k} \left( \delta +\gamma v{(dh, r)}- \beta u{(dh, r)} \right) v{(dh, r)}dr\nonumber \\{} & {} \quad - \sigma _2 \int _{ek}^{(e+1)k} v{(dh, r)} dB_{2}(r). \nonumber \\ F|_d^e(v)= & {} v{(dh, (e+1)k)}- v{(dh, ek)} \end{aligned}$$

48 $$\begin{aligned}{} & {} \quad - d_v k \left( \frac{v{\left( (d+1)h,(e+1)k\right) }-2v{(dh,(e+1)k)}+v{\left( (d-1)h,(e+1)k \right) }}{h^2} \right) \nonumber \\{} & {} \quad + k \left( \delta +\gamma v{(dh, ek)}- \beta u{(dh, ek)} \right) v{(dh, ek)} - \sigma _2 kv{(dh, ek)} dB_{2}(k). \end{aligned}$$

Hence, we conclude that

49 $$\begin{aligned}{} & {} E|F(v)|_d^e - F|_d^e(v)|^2 \nonumber \\{} & {} \quad \le 3d_v^2 E\left| \int _{ek}^{(e+1)k}\left( -v_{xx}(dh, r)+ \frac{v{\left( (d+1)h,(e+1)k\right) }-2v{(dh,(e+1)k)}+v{\left( (d-1)h,(e+1)k \right) }}{h^2} \right) dr\right| ^2 \nonumber \\{} & {} \qquad + 3E\left| \int _{ek}^{(e+1)k}\left( \left( \delta +\gamma v{(dh, r)}- \beta u{(dh, r)} \right) v{(dh, r)}-\left( \delta +\gamma v{(dh, ek)}-\beta u{(dh, ek)} \right) v{(dh, ek)}\right) dr\right| ^2\nonumber \\{} & {} \qquad + 3(\sigma _2)^2 \int _{ek}^{(e+1)k} E\bigg |\left( -v{(dh, r)} +v{(dh, ek)} \right) \bigg |^2dr. \end{aligned}$$

$E|F(v)|_d^e - F_d^e(v)|^2\rightarrow 0$ as $(d,e)\rightarrow \infty$. So, the given scheme for state variable *v* is consistent.

Similarly consistency for Eq. (7) is ,

50 $$\begin{aligned} F(w)|_d^e= & {} w{(dh, (e+1)k)}- w{(dh, ek)} - d_w \int _{ek}^{(e+1)k} w_{xx}(dh, r) dr \nonumber \\{} & {} \quad + \int _{ek}^{(e+1)k} \left( \rho - \eta u{(dh, r)} \right) w{(dh, r)}dr - \sigma _3 \int _{ek}^{(e+1)k} w{(dh, r)} dB_{3}(r). \nonumber \\ F|_d^e(w)= & {} w{(dh, (e+1)k)}- w{(dh, ek)} \end{aligned}$$

51 $$\begin{aligned}{} & {} \quad - d_w k \left( \frac{w{\left( (d+1)h,(e+1)k\right) }-2w{(dh,(e+1)k)}+w{\left( (d-1)h,(e+1)k \right) }}{h^2} \right) \nonumber \\{} & {} \quad + k \left( \rho - \eta u{(dh, ek)} \right) w{(dh,ek)} - \sigma _3 kw{(dh, ek)} dB_{3}(k). \end{aligned}$$

Hence, we conclude that

52 $$\begin{aligned}{} & {} E|F(w)|_d^e -F|_d^e(w)|^2 \nonumber \\{} & {} \quad \le 3d_v^2 E\left| \int _{ek}^{(e+1)k}\left( -w_{xx}(dh, r)+ \frac{w{\left( (d+1)h,(e+1)k\right) }-2w{(dh,(e+1)k)}+w{\left( (d-1)h,(e+1)k \right) }}{h^2} \right) dr\right| ^2 \nonumber \\{} & {} \qquad + 3E\left| \int _{ek}^{(e+1)k}\left( \left( \rho - \eta u{(dh, r)} \right) w{(dh, r)}-\left( \rho - \eta u{(dh, ek)} \right) w{(dh, ek)}\right) dr\right| ^2\nonumber \\{} & {} \qquad + 3(\sigma _3)^2 \int _{ek}^{(e+1)k} E\bigg |\left( -w{(dh, r)} +w{(dh, ek)} \right) \bigg |^2dr. \end{aligned}$$

$E|F(w)|_d^e -F|_d^e(w)|^2\rightarrow 0$ as $(d,e)\rightarrow \infty$. So, the given scheme for state variable *w* is consistent.  

### Theorem 4

The proposed SIFD scheme given in Eqs. (8)–(10) is consistent in the mean square with SPDE Eqs. (1)–(3).

### Proof

Now, the consistency of the proposed SIFD scheme for Eq. (8) is,

53 $$\begin{aligned} F(u)|_d^e= & {} u{(dh, (e+1)k)}- u{(dh, ek)}-\phi - d_u k u_{xx}(dh, r) \nonumber \\{} & {} \quad + k \left( \mu +\alpha v{(dh, r)}+ \xi w{(dh, r} \right) u{(dh, r)} - \sigma _1 ku{(dh, r)} dB_{1}(r). \nonumber \\ F|_d^e(u)= & {} u{(dh, (e+1)k)}- u{(dh, ek)}-u \end{aligned}$$

54 $$\begin{aligned}{} & {} \quad - d_u k \left( \frac{u{\left( (d+1)h,(e+1)k\right) }-2u{(dh,(e+1)k)}+u{\left( (d-1)h,(e+1)k \right) }}{h^2} \right) \nonumber \\{} & {} \quad + k \left( \mu +\alpha v{(dh, ek)}+ \xi w{(dh, ek)} \right) u{(dh, ek)} \nonumber \\{} & {} \quad - \sigma _1 ku{(dh, (e+1)k)} (B_{1((e+1)k)}-B_{1(ek)}). \end{aligned}$$

Hence, we conclude that $E|F(u)|_d^e -F|_d^e(u)|^2 \le$

55 $$\begin{aligned}{} & {} 3d_u^2 E\left| \int _{ek}^{(e+1)k}\left( -u_{xx}(dh, r)+ \frac{u{\left( (d+1)h,(e+1)k\right) }-2u{(dh,(e+1)k)}+u{\left( (d-1)h,(e+1)k \right) }}{h^2} \right) dr\right| ^2 \nonumber \\{} & {} \quad + 3E\left| \int _{ek}^{(e+1)k} \left( -\left( \mu +\alpha v{(dh, r)}+ \xi w{(dh, r)} \right) u{(dh, r)}+\left( \mu +\alpha v{(dh, ek)}+ \xi w{(dh, ek)} \right) u{(dh, ek)}\right) dr\right| ^2 \nonumber \\{} & {} \quad + 3(\sigma _1)^2 \int _{ek}^{(e+1)k} E\left| \left( -u{(dh, r)} +u{(dh, ek)} \right) \right| ^2dr. \end{aligned}$$

$E|F(u)|_d^e-F|_d^e(u)|^2\rightarrow 0$ as $(d,e)\rightarrow \infty$. So, the given scheme for state variable *u* is consistent.

Similarly, consistency for the Implicit finite scheme for equation (9).

56 $$\begin{aligned} F(v)|_d^e= & {} v{(dh, (e+1)k)}- v{(dh, ek)} - d_v \int _{ek}^{(e+1)k} v_{xx}(dh, r) dr \nonumber \\{} & {} \quad + \int _{ek}^{(e+1)k} \left( \delta +\gamma v{(dh, r)}\right) v{(dh, r)}dr-\int _{ek}^{(e+1)k}( \beta u{(dh, r)})v{(dh, r)}dr \nonumber \\{} & {} \quad - \sigma _2 \int _{ek}^{(e+1)k} v{(dh, r)} dB_{2}(r). \nonumber \\ F|_d^e(v)= & {} v{(dh, (e+1)k)}- v{(dh, ek)} \end{aligned}$$

57 $$\begin{aligned}{} & {} \quad - d_v k \left( \frac{v{\left( (d+1)h,(e+1)k\right) }-2v{(dh,(e+1)k)}+v{\left( (d-1)h,(e+1)k \right) }}{h^2} \right) \nonumber \\{} & {} \quad + k \left( \delta +\gamma v{(dh, ek)}\right) v{(dh, ek)}-k( \beta u{(dh, ek)}) v{(dh, (e+1)k)}\nonumber \\{} & {} \quad - \sigma _2 kv(dh, (e+1)k)) (B_{2((e+1)k)}-B_{2(mk)}). \end{aligned}$$

Hence, we conclude that

58 $$\begin{aligned}{} & {} E| F(v)|_d^e - F|_d^e(v)|^2 \nonumber \\{} & {} \quad \le 4d_v^2 E\left| \int _{ek}^{(e+1)k}\left( -v_{xx}(dh, r)+ \frac{v{\left( (d+1)h,(e+1)k\right) }-2v{(dh,(e+1)k)}+v{\left( (d-1)h,(e+1)k \right) }}{h^2} \right) dr\right| ^2 \nonumber \\{} & {} \qquad + 4E\left| \int _{ek}^{(e+1)k}\left( \left( \delta +\gamma v{(dh, r)} \right) v{(dh, r)}-\left( \delta +\gamma v{(dh, k)} \right) v{(dh, k)}\right) dr\right| ^2 \nonumber \\{} & {} \qquad +4E\left| \int _{ek}^{(e+1)k}\left( - \beta u{(dh, r)} v{(dh, r)} +\beta u{(dh, ek)} v{(dh,(e+1) k)}\right) dr\right| ^2 + 4(\sigma _2)^2 \int _{ek}^{(e+1)k} E\bigg |\left( -v{(dh, r)} +v{(dh, ek)} \right) \bigg |^2dr. \end{aligned}$$

$E|F(v)|_d^e-F|_d^e(v)|^2\rightarrow 0$ as $(d,e)\rightarrow \infty$. So, the given scheme for state variable *v* is consistent.

Similarly, consistency for the Implicit finite scheme for equation (10) is

59 $$\begin{aligned} F(w)|_d^e= & {} w{(dh, (e+1)k)}- w{(dh, ek)} - d_w \int _{ek}^{(e+1)k} w_{xx}(dh, r) dr \nonumber \\{} & {} \quad + \int _{ek}^{(e+1)k} \left( \rho w{(dh, r)}\right) dr - \int _{ek}^{(e+1)k} \left( \eta u{(dh, r)} w{(dh, r)}\right) dr \nonumber \\{} & {} \quad - \sigma _3 \int _{ek}^{(e+1)k} w{(dh, r)} dB_{3}(r). \nonumber \\ F|_d^e(w)= & {} w{(dh, (e+1)k)}- w{(dh, ek)} \end{aligned}$$

60 $$\begin{aligned}{} & {} \quad - d_v k \left( \frac{w{\left( (d+1)h,(e+1)k\right) }-2w{(dh,(e+1)k)}+w{\left( (d-1)h,(e+1)k \right) }}{h^2} \right) \nonumber \\{} & {} \quad + k \rho w{(dh, ek)} - k \eta u{(dh, ek)} w{(dh, (e+1)k)} \nonumber \\{} & {} \quad - \sigma _3 kw{(dh, ek)} (B_{3((e+1)k)}-B_{3(ek)}). \end{aligned}$$

Hence, we conclude that

61 $$\begin{aligned}{} & {} E| F(w)|_d^e-F|_d^e(w)|^2 \nonumber \\{} & {} \quad \le 4d_w^2 E\left| \int _{ek}^{(e+1)k}\left( -w_{xx}(dh, v)+ \frac{w{\left( (d+1)h,(e+1)k\right) }-2w{(dh,(e+1)k)}+w{\left( (d-1)h,(e+1)k \right) }}{h^2} \right) dr\right| ^2 \nonumber \\{} & {} \qquad + 4E\left| \int _{ek}^{(e+1)k} \left( \rho w{(dh, r)}-\rho w{(dh, ek)}\right) dr\right| ^2 \nonumber \\{} & {} \qquad + 4E\left| \int _{ek}^{(e+1)k} -\eta ( u{(dh, r)} w{(dh, r)}+ \eta (u{(dh, k)})w{(dh, (e+1)k)}dr\right| ^2 \nonumber \\{} & {} \qquad + 4(\sigma _3)^2 \int _{ek}^{(e+1)k} E\left| \left( -w{(dh, r)} +w{(dh, ek)} \right) \right| ^2dr. \end{aligned}$$

$E|F(w)|_d^e-F|_d^e(w)|^2\rightarrow 0$ as $(d,e)\rightarrow \infty$. So, the given scheme for state variable *w* is consistent.

## Convergence

The convergence of the stochastic implicit scheme is analyzed in the means square sense.

### Theorem 5

The stochastic implicit scheme is given by Eqs. (8)–(10) is convergent in the mean square sense.

### Proof

$$\begin{aligned} E\bigg |u_d^{e} -u\bigg |^2=E\bigg |(L_d^{e})^{-1}(L_d^{e}u_d^{e}-L_d^{e}u) \bigg |^2, \end{aligned}$$

as the scheme is consistent in the mean square sense i.e., $L_d^{e}u_d^{e} \rightarrow L_d^{e}u$ as $\Delta x \rightarrow 0, \Delta t \rightarrow 0$ and $(d\Delta x, e\Delta t,)\rightarrow (x,t)$,

$$\begin{aligned} E\bigg |(L_d^{e})^{-1}(L_d^{e}u_d^{e}-L_d^{e}u) \bigg |^2 \rightarrow 0, \end{aligned}$$

also, scheme is stable, then $(L_d^{e})^{-1}$ is bounded. So, $E\bigg |u_d^{e}-u \bigg |^2 \rightarrow 0$. Hence proposed scheme for *u* is convergent in the mean square sense. By doing the same practice, we can show that the scheme for *v*, *w* is convergent.

The convergence of another scheme can be deduced in a similar way.

## Graphically representation

The initial conditions are $u(x,0)=v(x,0)=w(x,0)=1$^43^.

The equilibria of given equations are

62 $$\begin{aligned}{} & {} E_1=\bigg (\frac{\phi }{\mu }, 0, 0\bigg ), \end{aligned}$$

63 $$\begin{aligned}{} & {} E_2=\bigg (\frac{\alpha \delta -\gamma \mu \mp \sqrt{(\gamma \mu -\alpha \delta )^2+ 4 \alpha \beta \gamma \phi }}{2\beta \alpha }, \frac{-(\delta \alpha +\gamma \mu ) \mp \sqrt{(\gamma \mu -\alpha \delta )^2+4 \alpha \beta \gamma \phi } }{2 \alpha \gamma },0\bigg ), \end{aligned}$$

64 $$\begin{aligned}{} & {} E_3=\bigg (\frac{\rho }{\eta }, 0, \frac{-(\mu \rho -\eta \phi ) }{\zeta \rho }\bigg ), \end{aligned}$$

65 $$\begin{aligned}{} & {} E_4= \bigg (\frac{\eta }{\rho }, \frac{-(\delta \eta -\beta \rho ) }{\gamma \eta }, \frac{\alpha \delta \eta \rho -\alpha \beta \rho ^2 +\gamma \eta ^2 \phi -\gamma \eta \mu \rho }{\eta \rho \gamma \zeta }\bigg ). \end{aligned}$$

Also, assume that $\varsigma _1=\frac{\mu \delta }{\beta }$, $\varsigma _2=\frac{\mu \rho }{\eta }$, $\varsigma _3=\varsigma _2\bigg [1+\frac{\alpha \delta }{\gamma \mu }\bigg (\frac{\varsigma _2}{\varsigma _1}-1\bigg )\bigg ]$ In^44^, Gazi et al. analyzed the different dynamics of the Fish farm model. They worked on the local behavior of the Fish farm system of the equations and summarized the existence of equilibria as follows

$$\begin{array}{ccc} \text{ Equilibrium } \text{ point } &{} \text{ feasibility } \text{ condition } &{} \text{ stability } \text{ condition } \\ E_1 &{} always &{} \phi<\varsigma _1,\phi<\varsigma _2 \\ E_2 &{} \phi>\varsigma _1 &{} \phi<\varsigma _3 \\ E_3 &{} \phi>\varsigma _2 &{} \varsigma _2<\varsigma _1\\ E_4 &{} \varsigma _2>\varsigma _1,\phi >\varsigma _3 &{} always \end{array}$$

By concluding this brief discussion, equilibrium $E_1$ is sable if $\phi<\varsigma _1,\phi <\varsigma _2$ and becomes unstable if exterior nutrients exceed the value of $\varsigma _1$. The equilibrium $E_2$ goes stable if exterior nutrient between the value of$\varsigma _1$ and $\varsigma _3$ with $(\varsigma _1<\varsigma _3)$. The equilibrium point $E_3$ is stable only if the feasibility condition, as well as stability condition, exists A. The given model is around the coexistence equilibrium if the exterior nutrient supply exceeds values $\varsigma _3$ with $\varsigma _1<\varsigma _2$. Thus, the given Fish form model is stable in various levels of exterior nutrient supply.

**Table 1:** The values of parameters.

$\mu$	$\alpha$	$\zeta$	$\delta$	$\gamma$	$\beta$	$\rho$	$\eta$
3	20	9	12	4	8	4	1.75

**Figure 1:**
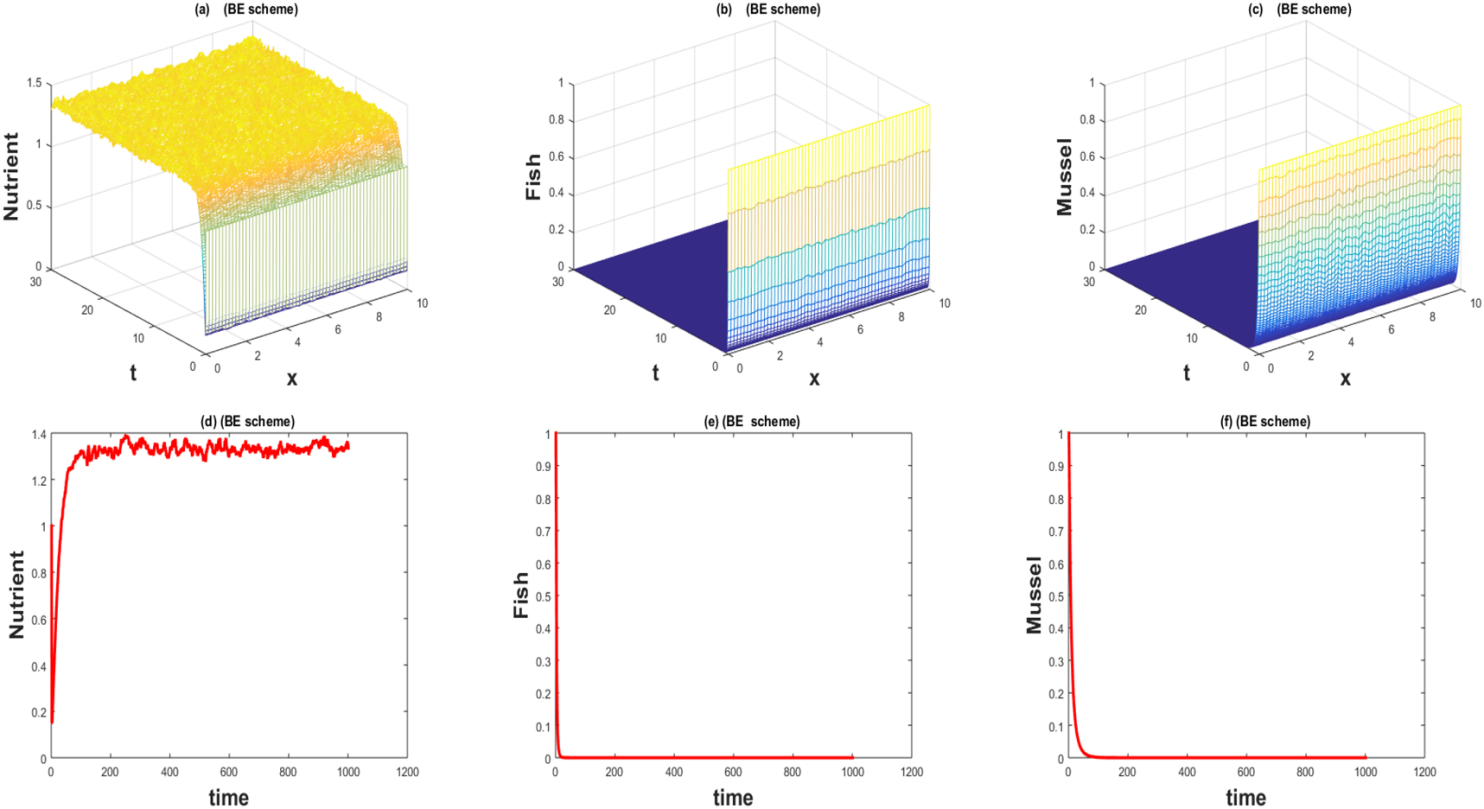
The 3D and 2D simulations of *u*, *v*, *w* for $\lambda _i=0.0216, \sigma _i=0.25, i=1,2,3$.

**Figure 2:**
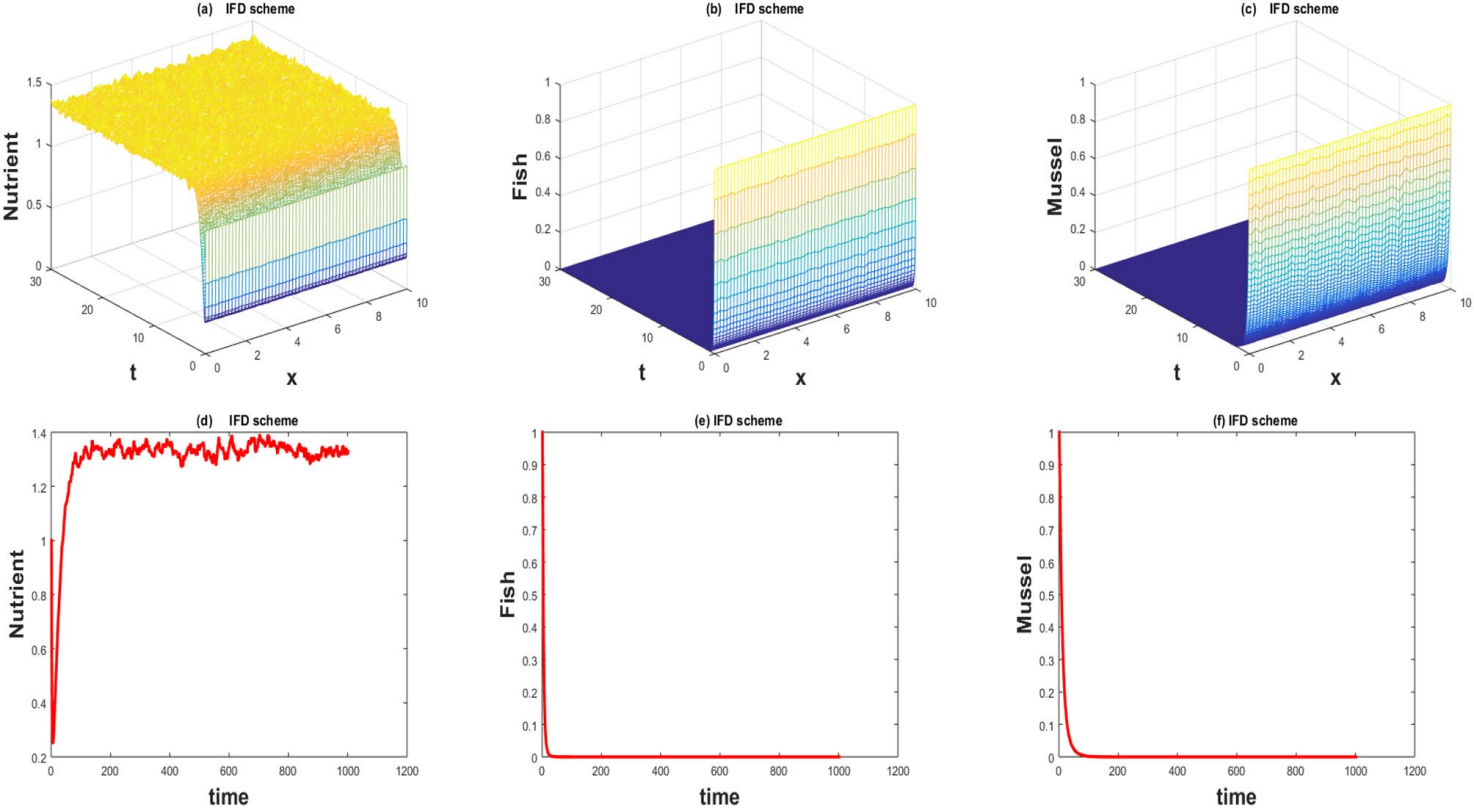
The 3D and 2D simulations of *u*, *v*, *w* for $\lambda _i=0.0216, \sigma _i=0.25, i=1,2,3$.

**Figure 3:**
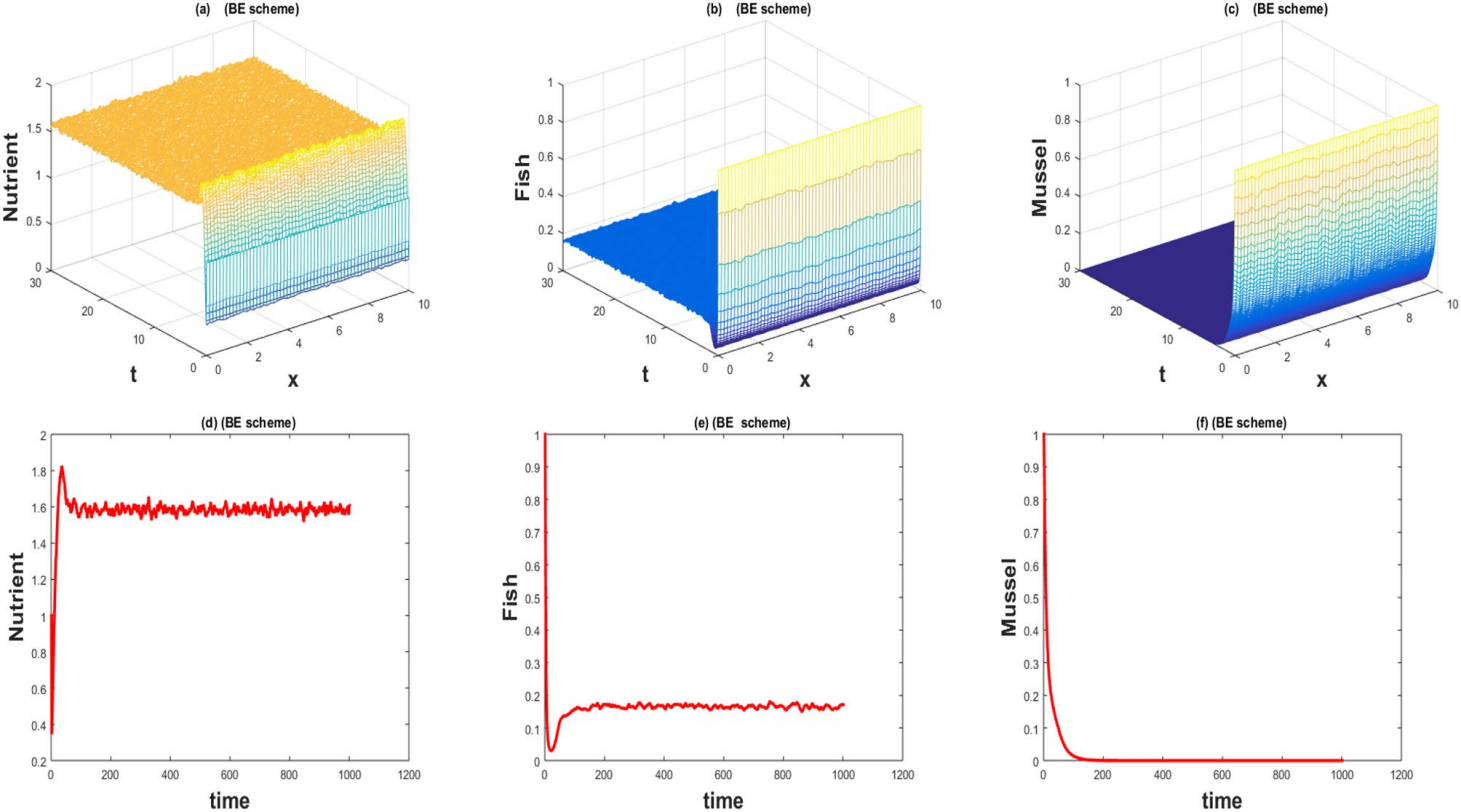
The 3D and 2D simulations of *u*, *v*, *w* for $\lambda _i=0.0108, \sigma _i=0.25, i=1,2,3$.

**Figure 4:**
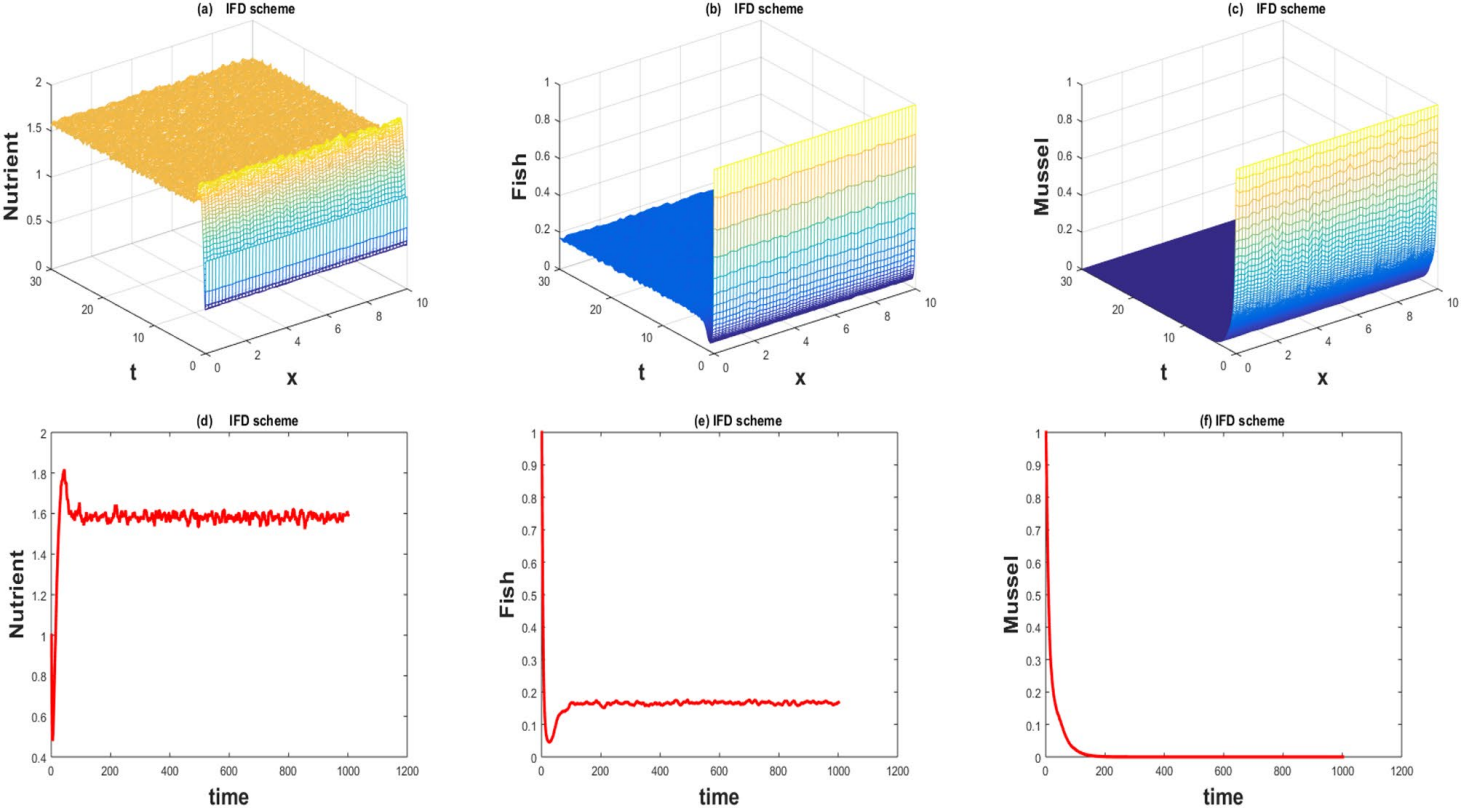
The 3D and 2D simulations of *u*, *v*, *w* for $\lambda _i=0.0108, \sigma _i=0.25, i=1,2,3$.

**Figure 5:**
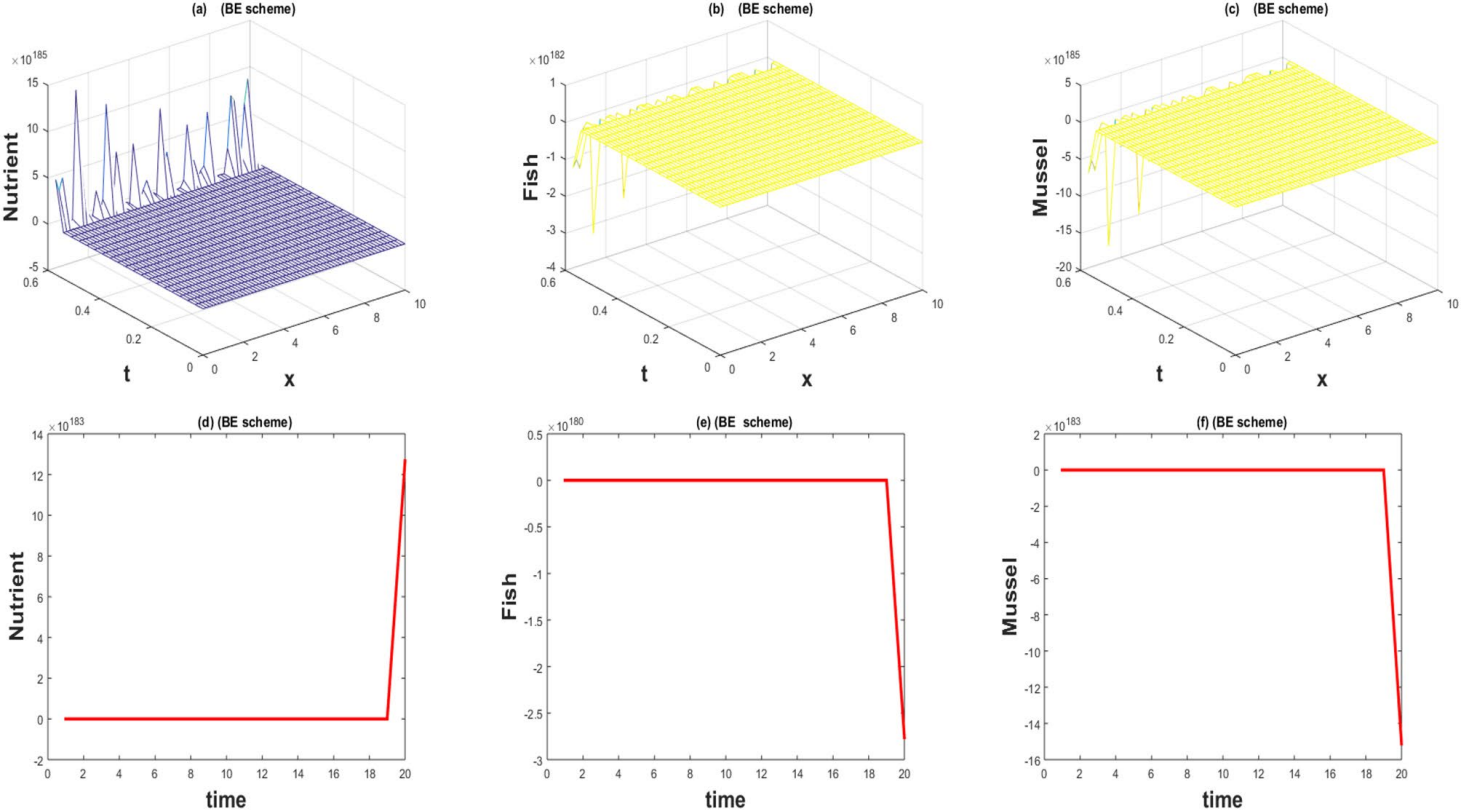
The 3D and 2D simulations of *u*, *v*, *w* for $\eta =10.75, \lambda _i=0.0108, \sigma _i=0.025, i=1,2,3$.

**Figure 6:**
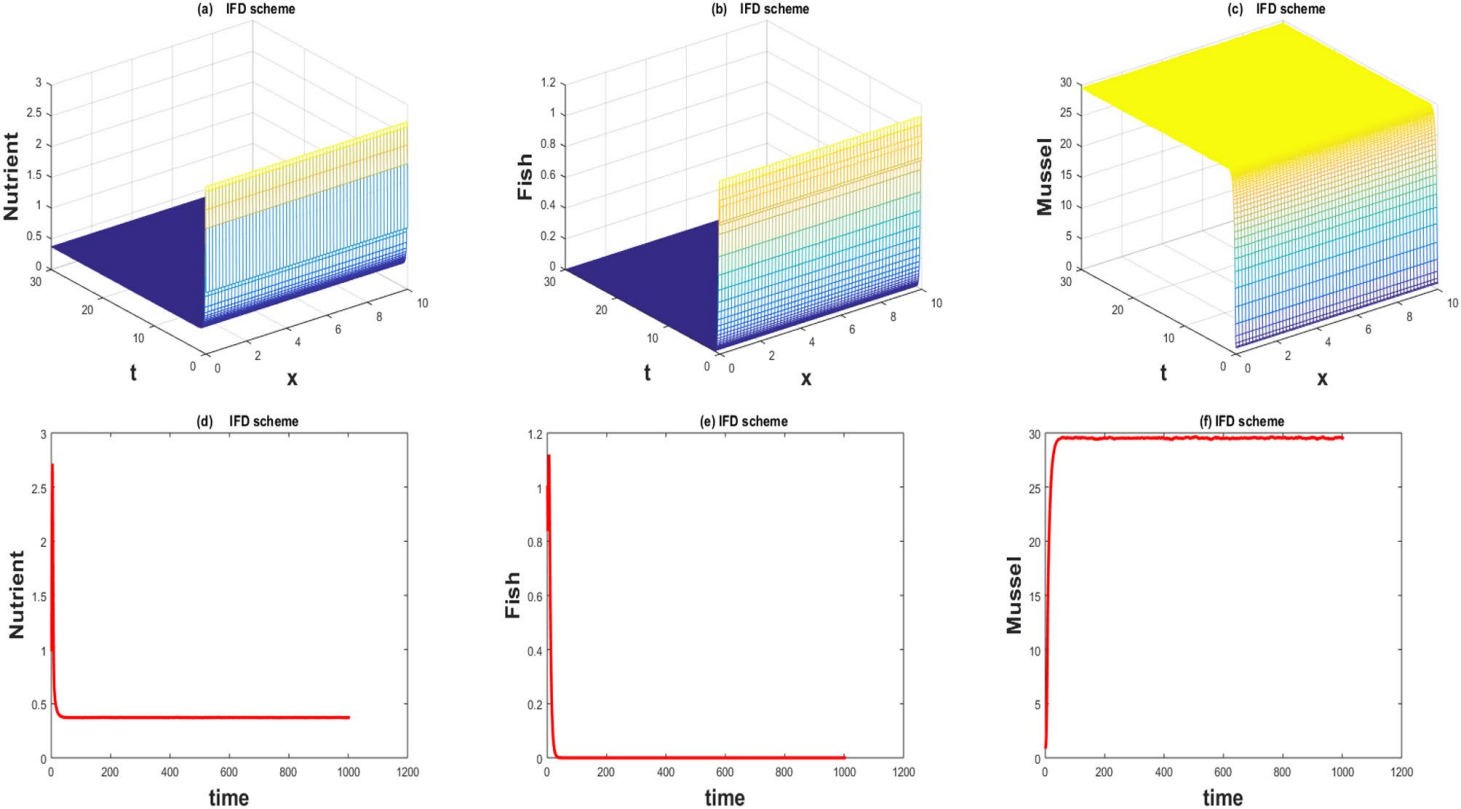
The 3D and 2D simulations of *u*, *v*, *w* for $\eta =10.75, \lambda _i=0.0108, \sigma _i=0.025, i=1,2,3$.

**Figure 7:**
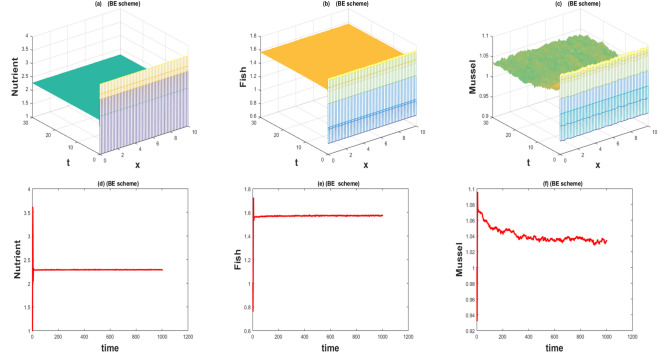
The 3D and 2D simulations of *u*, *v*, *w* for $\lambda _i=0.0108,i= \sigma _i=0.025, i=1,2,3$.

**Figure 8:**
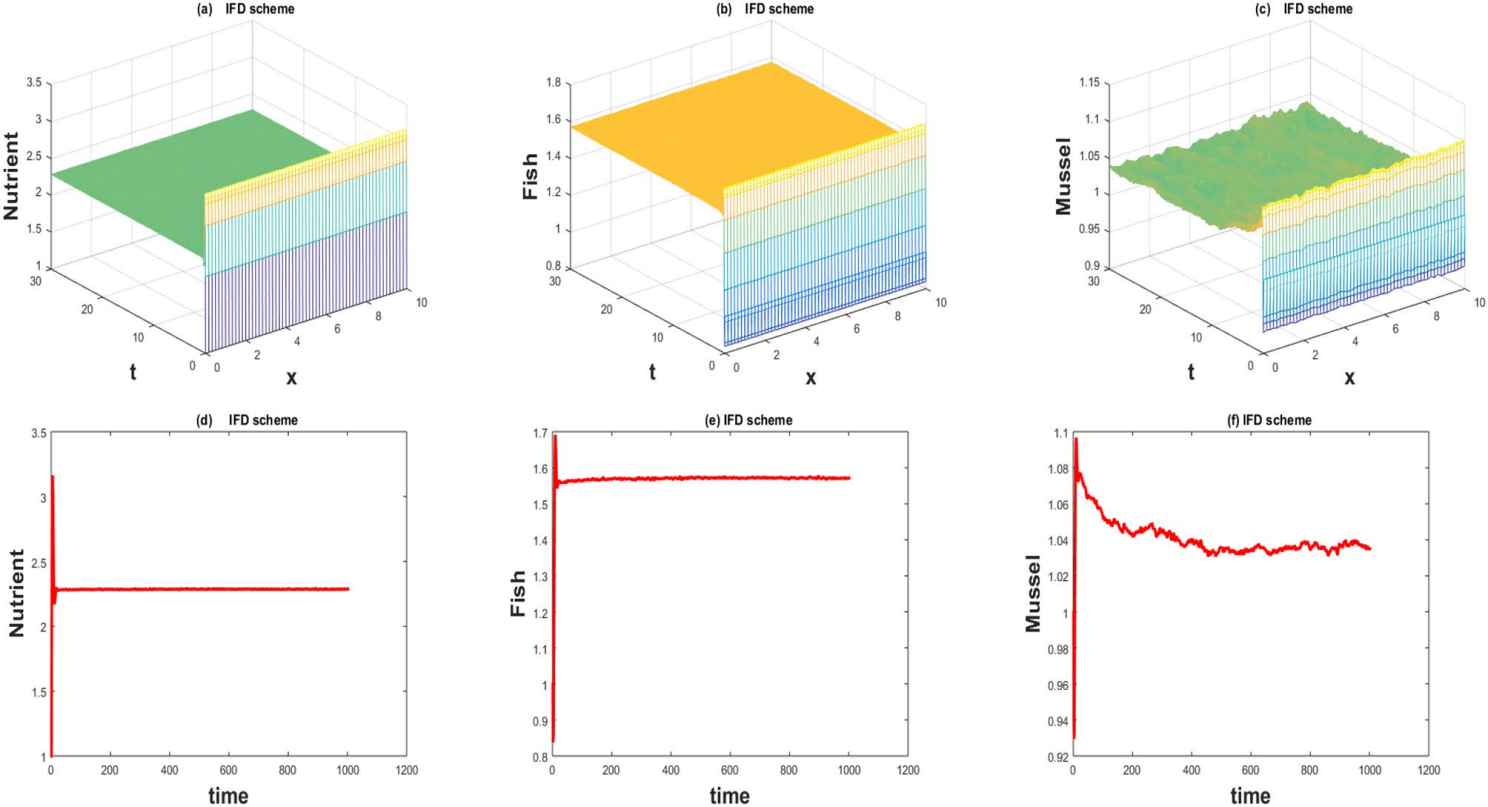
The 3D and 2D simulations of *u*, *v*, *w* for $\lambda _i=0.0108, \sigma _i=0.25, i=1,2,3$.

The values of the parameters that are given in Table 1 showed by the Figs. 1, 2, 3, 4, 5, 6, 7 and 8) . The Figs. 1, 2, 3 and 4) have noise strength 0.25 and the Figs. 4, 5, 6, 7 and 8 have noise strength 0.025. The BFD Scheme for external nutrient $\phi =4$ is discussed in the Fig. 1 and $E_1$ is a stable point nevertheless the graphical representation displays that state variable *u*(*x*, *t*) and *v*(*x*, *t*) have negative values and it is an imperfection in SBE scheme. Furthermore, the SIFD scheme for external nutrient $\phi =4$ discussed in the Fig. 2 and $E_1$ is a stable point nevertheless the graphical representation displays that state variable *u*, *v*, and *w* have positive values for the entire domain and this showed that this scheme is stable as well as keep the entire behavior. The SBE scheme for external nutrient $\phi =10$ is discussed in the Fig. 3 and $E_2$ is the stable point but the graphical representation displays that the state variable *u*(*x*, *t*) has negative values and it is insignificant for the population dynamics. Similarly, The IFD scheme for external nutrient $\phi =10$ discussed in the Fig. 4 and $E_2$ is a stable point nevertheless the graphical representation displays that entire state variable *u*, *v*, and *w* have positive values. Moreover, The BFD scheme for external nutrient $\phi =100$ is discussed in Fig. 5 and $\eta =10.75$, is not stable and the state variables have negative values along with divergent behavior. In addition, The IFD scheme for external nutrient $\phi =100$ discussed in the Fig. 6 and $\eta =10.75$, the equilibrium point $E_3$ is stable as well as its graphical behavior display that all state variables own the positivity. The BFD scheme for external nutrient $\phi =100$ is discussed in Fig. 7$E_4$ is gained and the state variables keep the positivity. Similarly, The IFD scheme for external nutrient $\phi =100$ discussed in the Fig. 8 and the $E_4$ point is stable nevertheless the graphical representation displays that the entire state variable has positive values. MATLAB 2015*a* is manipulated for this discussion and analysis of the stated Fish Form model.

The fish farm models are population dynamics and necessarily their; solutions must be positive. We have used 2 schemes for the numerical approximation of the governing model. One technique fails to preserve the positive behavior while the other has positive and converges towards the true steady states. One of the most compelling reasons to consider this model with a numerical scheme is to construct and apply the scheme in a way that yields positive solutions. As the governing model is population dynamics and its minimum values can be zero but can never be negative. So its solution must preserve the positivity. The results of the stochastic implicit finite difference scheme resemble the positive steady states. As population dynamics have random behavior, it is better to use the continuous model with a random effect. Such random behavior is observed in every physical phenomenon at a certain level. So we incorporate diffusion as well as random behavior in the underlying model.

## Conclusions

The fish farm model under the influence of the Wiener process is considered. The literature on numerical study for the stochastic partial differential equations is required. We have designed two novel and time-efficient numerical techniques for the computational study of the underlying model. The schemes are proposed stochastic backward Euler(SBE) and proposed stochastic Implicit finite difference(SIFD) scheme. The analysis of schemes is analyzed in the mean square sense. Both schemes are compatible with the given system of equations and the linear analysis of schemes is analyzed. The underlying model has four equilibrium points. When the SBE scheme is used for the study, equilibrium $E_1$ is successfully gained but it also shows negative behavior, $E_2$ is also obtained with negative behavior, $E_3$ is not gained because it has divergent behavior, and $E_4$ is obtained with positive behavior. On the other hand, when the SIFD scheme is applied, equilibrium point $E_1$ is successfully attained with positive behavior $E_2$ is attained with convergent and positivity, $E_3$ also gained with positive behavior, and $E_4$ is successfully obtained with positive behavior for the given values of the parameter. From the graphical behavior of the system, it is observed that the effect of external food is the main factor that controls the dynamics of the model. The simulations are drawn for various values of the parameters.The study of the SPDEs and their dynamics is the need of the hour. So, we will try to analyze its various aspects such as qualitative analysis of the model and use of optimal strategies to control the SPDEs.

## Data Availability

Data will be provided by corresponding author on reasonable request.
